# Supplementary material for: Auditory cortical activity drives feedback-dependent vocal control in marmosets
Source: Nat Commun. 2018 Jun 29;9:2540. doi: 10.1038/s41467-018-04961-8 (PMC6026141; doi:10.1038/s41467-018-04961-8)
Supplement: Supplementary file 1 — Supplementary Information [file 41467_2018_4961_MOESM1_ESM.pdf]

Supplementary Information

Auditory cortical activity drives feedback-dependent vocal control in marmosets

Eliades et al.

Supplementary Note 1

Details regarding sample sizes, p-values, and statistical tests of individual figure panels are detailed blow.

Fig. 1d. All n values represent number of vocalizations. Analyses were parametric ANOVAs with Bonferroni corrections. Expected (mean) frequency changes during normal conditions were subtracted from individual samples prior to averaging.

m96z trill: n=[7513, 4074, 3976] (normal/-2/+2); means: 0± 36.8, 30.2±42.4, -38.4±35.0 (mean±sd); F=33.08, p=4.6E-15, all pairwise p <0.001.

m96z trillphee: n=[3528, 1939, 2600]; means: 0±27.9, 84.3±34.9, 046,1±33.7; F=95.52, p=9.99E-42, all pairwise p<0.001.

mm058 trill: n= [1415, 1004, 504]; means: 0±27.9, 84.1±30.5, -54.3±29.1; F=44.30, p=1.12E-19, all pairwise p<0.001.

mm058 trillphee: n=[1389, 710, 1152]; means: 0±27.6, 63.0±36.4, -42.1±34.5; F=23.45, p=7.78E-11, all pairwise p<0.001 save normal vs. +2 where p=0.003.

Fig 1e. Vocal sample sizes: normal: 11,159, -2ST: 11,984, +2ST: 11,533.

Fig 2a. Firing rates -10.2±6.4 spk/s (mean±sd, normal), -9.5±6.7 (amplified), -6.5±4.8 (+2ST). N=355 (vocalizations), Chi-squared=26.6, p=1.7E-6 (Kruskall-Wallis), pairwise comparisons p-values: 0.43 (normal vs. amp), <0.001 (norm vs +2), <0.001 (amp vs. +2).

Fig. 2b. Number of units +2ST: 608, -2ST: 638

Fig. 3a. Firing rates -8.8±6.9 spk/s (normal), -7.8±7.0 (amplified), -4.4±6.2 (-2ST). N=455 (vocalizations), Chi-squared=28.0, p=8.5E-7 (Kruskall-Wallis), pairwise comparisons p-values: 0.63 (normal vs. amp), <0.001 (norm vs -2), <0.001 (amp vs. -2).

Fig 3c. Pearson correlation coefficient r=0.624, p<0.001 (N=28 vocalizations), Bottom:

Fig 3d. Pearson correlation coefficients, p<0.05 indicated (N=88 normal, 233 amplified, 136 -2ST).

Fig 4a. Indicated N values are number of units. Indicated values for p<0.05 (Wilcoxon signed-rank tests with FDR corrections).

Fig. 4b. Unit numbers as in Fig4a. Shaded significance (p<0.05) for individual units (Wilcoxon signed-rank). Mean and standard deviation are indicated.

Fig. 4c & d. Indicated values for p<0.05 as in Fig 4a.

Fig. 5. N=479 units (-2ST), 512 (+2ST) . Indicated values for p<0.05 (from Pearson correlations with FDR corrections).

Fig. 6a. +2ST RMI change: 0.24±0.31, mean±std; N=638, z=23.3, p<0.001, Wilcoxon signed-rank. -2ST: 0.27±0.37; N=608, z=23.8, p<0.001. N is number of units.

Fig. 6b. Number of units as in Fig 6a. Pearson correlations: +2ST (r=-0.53, p<0.001) and -2ST (r=-0.53, p<0.001).

Fig. 6c. Number of units as in Fig 6a. Pearson correlations: +2ST (r=-0.02, p=0.65) and -2ST (r=0, p=0.97).

Fig. 6d. Pearson correlations for absolute value vocal correlation coefficients vs. RMI: r=-0.02 (p=0.41) for all data, and r=-0.04 (p=0.77) for only significant units.

Fig. 6g. Number of units as in Fig 6a. Left hemisphere change: 0.26±0.40, Right hemisphere: 0.30±0.44 (z=2.40, p=0.016, Wilcoxon rank-sum).

Fig. 6h. Number of units as in Fig 6a. Left hemisphere correlation: 0.19±0.17, Right hemisphere: 0.20±0.44 (z=0.21, p=0.84, Wilcoxon rank-sum).

Fig. 7b. Median 38.5ms, IQR: 29.6-51.7ms

Fig. 7f. P-values for individual plots determined using Wilcoxon signed-rank tests for each electrode comparing median frequency changes from baseline.

Fig. 8. P-values for individual plots determined using Wilcoxon signed-rank tests for each electrode comparing median frequency changes from baseline.

Fig. 9a. N=32 electrodes (left), 46 (right). z=2.57, p=0.010 (Wilcoxon rank-sum between hemispheres). Multivariate linear regression using factors: animal, hemisphere, row, and column showed overall R^2=0.12, F=2.54, p=0.047. Individual p-values were, respectively: 0.38, 0.004, 0.46, and 0.48.

Fig. 9b. N as in Fig.9a. Total: r=0.36, p=0.001 (Spearman), LH: r=0.07, p=0.70, RH: r=0.41, p=0.005.

Fig. 9c. N as in Fig.9a. Total: r=-0.37, p=0.001 (Spearman), LH: r=-0.27, p=0.14, RH: r=-0.36, p=0.016.

Fig. 9d. N as in Fig.9a. Total: $r=0.38$, $p=0.001$ (Spearman), LH: $r=0.28$, $p=0.13$, RH: $r=0.34$, $p=0.022$.

Fig. 9e. Trill mean frequency: 6.95±0.80 kHz (mean±sd). Shaded area is 95% confidence interval for shuffled estimation of CF tuning (see Methods).
